# Examining the Relationship between *Toxoplasma gondii* and Seropositivity and Serointensity and Depression in Adults from the United Kingdom and the United States: A Cross-Sectional Study

**DOI:** 10.3390/pathogens10091101

**Published:** 2021-08-29

**Authors:** Shawn D. Gale, Lance D. Erickson, Bruce L. Brown, Dawson W. Hedges

**Affiliations:** 1 Department of Psychology, Brigham Young University, Provo, UT 84602, USA; bruce_brown@byu.edu (B.L.B.); dawson_hedges@byu.edu (D.W.H.); 2 The Neuroscience Center, Brigham Young University, Provo, UT 84602, USA; 3 Department of Sociology, Brigham Young University, Provo, UT 84602, USA; lance_erickson@byu.edu

**Keywords:** *Toxoplasma gondii*, depression, affective disorders, UK Biobank

## Abstract

Infecting approximately one-third of the world’s population, the neurotropic protozoan *Toxoplasma gondii* has been associated with cognition and several neuropsychiatric diseases including schizophrenia and bipolar disorder. Findings have been mixed, however, about the relationship between *Toxoplasma gondii* and depression, with some studies reporting positive associations and others finding no associations. To further investigate the association between *Toxoplasma gondii* and depression, we used data from the UK Biobank and the National Health and Examination Survey (NHANES). Results from adjusted multiple-regression modeling showed no significant associations between *Toxoplasma gondii* and depression in either the UK Biobank or NHANES datasets. Further, we found no significant interactions between *Toxoplasma gondii* and age, sex, educational attainment, and income in either dataset that affected the association between *Toxoplasma gondii* and depression. These results from two community-based datasets suggest that in these samples, *Toxoplasma gondii* is not associated with depression. Differences between our findings and other findings showing an association between *Toxoplasma gondii* and depression could be due to several factors including differences in socioeconomic variables, differences in *Toxoplasma gondii* strain, and use of different covariates in statistical modeling.

## 1. Introduction

*Toxoplasma gondii* is an intracellular apicomplexan protozoon that infects an estimated one-third of the world’s population [1]. While members of the cat family are the definitive host of *Toxoplasma gondii*, humans can become infected from ingesting *Toxoplasma gondii* oocysts in infected undercooked meat, from exposure to cat feces, or from transmission during pregnancy [2]. Because *Toxoplasma gondii* is neurotropic with lifelong persistence in the brain of the host [3], it is possible that it affects brain function, and, indeed, many studies have reported associations between acute and latent infection with *Toxoplasma gondii* and behavior [4] and cognitive function in humans [5]. In addition, infection with *Toxoplasma gondii* might be associated with abnormalities in brain structure [6], and the intraneuronal cysts that *Toxoplasma gondii* forms might affect brain dopamine, glutamate, serotonin, and gamma aminobutyric acid [7], as well as alter gene expression [8].

*Toxoplasma gondii* has been associated with deficits in cognitive function in humans in many [9,10,11,12,13] but not all [14,15,16] studies, with dementia [17,18], and with epilepsy [19]. Although not all findings have been consistent [20], *Toxoplasma gondii* has been associated with several neuropsychiatric disorders including schizophrenia [21], obsessive-compulsive disorder [4], bipolar disorder, and substance abuse [22]. However, the results of studies investigating associations between *Toxoplasma gondii* and depressive disorders have been mixed. Some studies have found associations between *Toxoplasma gondii* and depressive disorders [23,24,25,26,27,28], whereas others have not [29,30,31,32,33].

Differences between the findings of previous studies investigating the association between *Toxoplasma gondii* and depression could be due to a variety of factors, including differences in sample size and inclusion of different covariates and different numbers and types of covariates in the statistical models used in these studies, resulting in differing amounts of control over possible confounding. In addition, systematic differences in exposure to stress, adversity, socioeconomic status, and infection in early life between samples could affect response to infection later in life [34], thus potentially altering associations between *Toxoplasma gondii* and depression depending on characteristics of individual study samples.

These inconsistent findings in studies investigating the association between *Toxoplasma gondii* and depressive disorders and a lack of consensus regarding causes of the inconsistencies create a need for further exploration of this relationship. Therefore, we replicate previous studies of the relationship between *Toxoplasma gondii* and depression and further characterize the relationship using two large community samples, one from the United Kingdom and another from the United States. Additionally, we adjust the estimated relationship for several potentially confounding biological, medical, and socioeconomic variables to obtain more accurate estimates of the relationship between *Toxoplasma gondii* and depressive disorders. Moreover, each of the two samples we used assessed depression using different methods, providing a broad assessment of depression.

## 2. Results

Table 1 shows the demographics and other characteristics of both the UK Biobank and the NHANES. With the temporal referent in the UK Biobank sample being lifetime experience, 27 percent experienced depression, while only six percent of the NHANES sample had depression with its temporal referent being the previous two weeks. The seroprevalence of *Toxoplasma gondii* in the UK Biobank sample was 29 percent; in the NHANES, the seroprevalence was 17 percent. The average age in the UK Biobank sample was 57.06 years, and it was 54.49 years in the NHANES sample. Fifty-five percent of the UK Biobank sample were women, and 53 percent of the NHANES sample were women. 

Based on the results of adjusted multivariable regression modeling, there were no significant associations between *Toxoplasma gondii* seropositivity and depression in either the UK Biobank or NHANES datasets (Table 2) (Figure S1). Further, there were no significant interactions in adjusted models between *Toxoplasma gondii* seropositivity and age (Figure S2a), sex (Figure S2b), educational attainment (Figure S2c), and income (Figure S2d) in predicting depression (Table 3). In supplemental analyses, we estimated the NHANES models from Table 2 and Table 3 using the full NHANES sample, including in these analyses all participants from ages 18 to 80 years who had data for *Toxoplasma gondii*, depression, and the relevant covariates (N = 5028). In these analyses, there also were no significant associations between *Toxoplasma gondii* seropositivity and depression (Table S1), nor were there any significant interactions of *Toxoplasma gondii* with age, sex, educational attainment or income in the full NHANES (Table S2). 

## 3. Discussion

The main findings from this study are that there were no associations between *Toxoplasma gondii* and depression in either the community-based sample from the UK or in the community-based sample from the US. Even in the expanded NHANES sample of 5028 that included participants ranging in age from 18 to 80 years, there were no significant associations between *Toxoplasma gondii* seropositivity and depression. Similarly, there were no significant interactions predicting depression between *Toxoplasma gondii* and age, sex, educational attainment, and income in either dataset, suggesting that none of these variables is related to vulnerability for depression in people seropositive for *Toxoplasma gondii* in the UK Biobank and NHANES samples. Much of the difference in depression prevalence between the two datasets is most likely due to how depression was assessed in the datasets, with the UK Biobank assessing lifetime prevalence of depression and the NHANES assessing point prevalence of depression. Overall, these findings from two large datasets from high-income nations do not show significant associations between *Toxoplasma gondii* and depression.

The lack of an association that we found is consistent with several previous studies that also found no associations between *Toxoplasma gondii* and depressive disorders [29,30,31,32,33]. Both Pearce et al. [33] and Gale et al. [32] used earlier data cycles from the NHANES. Although Pearce et al. [33] found an association between bipolar disorder and *Toxoplasma gondii*, neither Pearce et al. [33] nor Gale et al. [32] found associations between *Toxoplasma gondii* and depression. In contrast, our results differ from other studies that did find associations between *Toxoplasma gondii* and depression [24,25,26,28].

The differences in results of the studies that have investigated associations between *Toxoplasma gondii* and depression could be due to several factors, which could help identify reasons about how *Toxoplasma gondii* might be associated with depression in some samples but not in others. In particular, there seem to be geographic patterns among samples that identify a relationship between *Toxoplasma gondii* and those that do not. A possible general trend suggests that studies from lower-income countries [25,26,35] report associations between *Toxoplasma gondii* and depression. In contrast, studies from higher income nations [32,33] do not find associations between *Toxoplasma gondii* and depression. However, there are exceptions [23] to this overall pattern. 

The geographic differences in findings could be caused by differences in nutrition, early-life history, or socioeconomic factors that are systematically different in these locales. In this regard, Nasirpour et al. [26] report an important finding showing that factors such as nutritional status might affect the association between *Toxoplasma gondii* and depressive disorder. Likewise, in their meta-analysis, Sutterland et al. [22] report that associations between *Toxoplasma gondii* and schizophrenia might be stronger in Africa, Asia, the Middle East, and South America than in Europe and North America. Differences in early-life history and socioeconomic factors, as well as in strain of *Toxoplasma gondii*, therefore, could account for some of the differences between their findings and ours. In this regard, it is possible that early-life adversity and socioeconomic factors might be associated with proinflammatory states in adulthood and altering of the immune response to the effects of infectious diseases later in life [34], which could affect associations between *Toxoplasma gondii* and depression depending upon geographical regions.

Considering the possible geographic patterns in findings and the variation in the distribution of strains of *Toxoplasma gondii* across the world [36], it could be that the different strains of *Toxoplasma gondii* could affect associations between *Toxoplasma gondii* and depressive disorders differently. In this regard, Xiao et al. [37] found that the type I strain was associated with psychosis and affective psychosis in offspring of mothers seropositive for *Toxoplasma gondii*, whereas types II and III were not. While we did not have data about the particular *Toxoplasma gondii* strains in the UK Biobank and in the NHANES data, we agree with previous studies evaluating associations between *Toxoplasma gondii* and depression that additional research considering individual strains of *Toxoplasma gondii* is needed to determine whether some strains might be associated with enhanced vulnerability to depression. However, the findings of no associations between *Toxoplasma gondii* and depression in the samples we used from the UK and the US suggest that the predominant strains in those regions are not associated with depression. 

Host genetic factors also might be involved in determining whether *Toxoplasma gondii* is associated with depression. In a male-rat model, for example, infection with *Toxoplasma gondii* increased anxiety-like behavior but only increased depressive-like behavior in the rat strain already at risk for depressive-like behavior, even though infection with *Toxoplasma gondii* increased cytokine expression in all rats [38]. These findings indicate that better identification of host genetic risk factors for depression might further clarify the association between *Toxoplasma gondii* and depression. 

In addition to factors such as differences in strain, socioeconomic variables, and exposure to early-life adversity, inclusion of different covariates in the statistical modeling across studies could also affect associations between *Toxoplasma gondii* and depressive disorders, necessitating further research investigating environmental including early-life exposure influences on depression. 

While our study has several strengths including two different datasets from two nations, large sample sizes, inclusion of covariates that could possibly confound the association between *Toxoplasma gondii* and depression, and inclusion of interaction terms in the statistical modeling, several factors and limitations require consideration when interpreting its findings. The study design is cross-sectional and not longitudinal. As such, we do not have information about when the initial exposure to *Toxoplasma gondii* occurred. It is possible that exposure at different developmental periods could have different effects. In addition, we do not know which *Toxoplasma gondii* strain infected the participants in the study, which limits comparison with findings from other geographical regions. Although we attempted to control for possible confounding variables by including several covariates into our models that could potentially affect associations between *Toxoplasma gondii* and depression, there is still the possibility of residual confounding. Because of the characteristics of the particular datasets we used for our study, the generalizability of these findings to other regions of the world is limited. 

In conclusion, using two large, well-defined, community-based datasets from the UK and from the US, we found no associations between *Toxoplasma gondii* seropositivity and serointensity and depression in models adjusted for several possibly confounding variables. In addition, there were no significant interactions between *Toxoplasma gondii* and age, sex, educational attainment, and income in predicting depression. However, considerable research remains to be done in identifying factors that could account for the differences in the results of our study and similar studies finding no associations between *Toxoplasma gondii* and depression and the results of other studies that have found such associations. 

## 4. Materials and Methods

### 4.1. Study Samples

The data we used for this study come from the UK Biobank and from the National Health and Nutrition Survey (NHANES) of the United States’ Centers for Disease Control and Prevention.

#### 4.1.1. UK Biobank

The UK Biobank contains demographic and medical data from approximately 500,000 community-dwelling adults collected at 22 centers in the United Kingdom. Participants in the UK Biobank were enrolled between ages 40 and 70 years between 2006 and 2010, and all provided informed consent (http://biobank.ctsu.ox.ac.uk/crystal/field.cgi?id=200 (accessed on 7 February 2021)). The UK Biobank received regulatory approval (reference 11/NW/0382) to collect a broad range of demographic and medical data via questionnaires, nurse interviews, and laboratory studies (http://www.ukbiobank.ac.uk (accessed on 7 February 2021)) [39]. Although the UK Biobank is not designed to be representative of the UK population, it can be used to investigate associations between exposures and outcomes (http://www.ukbiobank.ac.uk/wp-content/uploads/2017/03/access-matters-representativeness-1.pdf (accessed on 7 February 2021)). In our study, we included all participants who had serological data for exposure to *Toxoplasma gondii* and data for depressive disorders (N = 2271). We obtained approval from the UK Biobank to conduct this research (http://biobank.ctsu.ox.ac.uk/crystal/field.cgi?id=200 (accessed on 7 February 2021)). 

#### 4.1.2. NHANES

The NHANES is a publicly available dataset accessible without application. We used the anonymized data from the 2013–2014 cycle, which was the most recent NHANES cycle that contained the variables needed to evaluate the association between *Toxoplasma gondii* and depression. We included participants who had data for *Toxoplasma gondii* and depression who were between the ages of 40 and 70 years so that the age distribution of the NHANES data (N = 2484) would match the UK Biobank data. Although the NHANES uses a complex sampling design and requires statistical weighting to render the results of statistical analyses representative of the US population, the assays for *Toxoplasma gondii* were done on leftover serum samples rendering statistical weighting unnecessary. Because we did not use statistical weighting, however, our results are not representative of the US population.

### 4.2. Assessment of Depression

For the UK Biobank data, we utilized the methods employed by Smith et al. [40], which were developed to determine whether a given participant had a lifetime history of major depression. The questions used to identify major depression included items from the Patient Health Questionnaire [41], questions related to seeking treatment for mood-related symptoms, and items from a screening measure given to potential UK Biobank participants at baseline (https://biobank.ctsu.ox.ac.uk/showcase/showcase/docs/TouchscreenQuestionsMainFinal.pdf (accessed on 7 February 2021)). Thus, Smith et al. [40] developed criteria to identify probable recurrent major depression, moderate, a single episode of major depression, and no major depression. To match the data available in NHANES (see below), we recoded a single episode of major depression, probable recurrent major depression (moderate), and probable recurrent major depression (severe) as depression. Indicators of probable bipolar disorder were also available, but we excluded those cases from the analytic sample because there were not enough positive cases who were also tested for *Toxoplasma gondii* to carry out an analysis.

The NHANES used the Patient Health Questionnaire-9 (PHQ-9) to evaluate depressive symptoms [42,43] during a computer-assisted interview. Details of how this measure was coded and a list of the specific questions on this measure can be found at (https://wwwn.cdc.gov/Nchs/Nhanes/2013-2014/DPQ_H.htm-Component_Description (accessed on 7 February 2021)). The PHQ-9 is a self-report measure that asks about the frequency of depressive symptoms during the previous two weeks. Each question is rated from 0 to 3 with 0 being “not at all” and 3 representing “nearly every day” for a possible range on this measure between 0 and 27. In addition to the nine assessment questions, participants were also asked to rate “How difficult have these problems made it for you to do your work, take care of things at home, or get along with people.” Thus, this last question provided information regarding level of functional impairment associated with the depressive symptoms. This last, non-scored question, ranged from 0, “not at all difficult,” to 3, “extremely difficult.” To be coded as depressed, participants had to meet all PHQ-9 criteria. Namely, they had to report 2 or 3 for at least one of the questions about whether they felt little interest in doing things or that asked if they felt down, depressed, or hopeless; they also had to indicate a 2 or 3 for five of the nine total symptom questions and had to indicate that the depressive symptoms made activities of daily life at least somewhat difficult.

### 4.3. Toxoplasma gondii

To determine exposure to *Toxoplasma gondii*, the UK Biobank measured IgG antibody concentrations against sag1 and p22 antigens [44]. Detailed methodology regarding the use of median fluorescence intensities in the validation of multiplex serology techniques carried out on the sera are available in the UK Biobank; determination of cut-offs, sensitivity and specificity, and other information regarding determination of *Toxoplasma gondii* seropositivity can be found elsewhere [44]. If respondents’ antigens were greater than 100 for p22 or greater than 160 for sag1, they were considered *Toxoplasma gondii* seropositive (https://biobank.ctsu.ox.ac.uk/crystal/field.cgi?id=23062 (accessed on 7 February 2021), see also https://biobank.ctsu.ox.ac.uk/crystal/crystal/docs/infdisease.pdf (accessed on 7 February 2021)). In addition to seropositivity, we also evaluated serointensity, or concentration of the antibodies. We included the natural log of p22 and sag1values as continuous serointensity measures in separate analyses and included the mean of the standardized natural log of p22 and sag1 values as a final continuous measure of *Toxoplasma gondii* serointensity in the UK Biobank data.

In the NHANES dataset, respondents were considered seropositive if anti-*Toxoplasma gondii* IgG antibodies from the enzyme immunoassay were greater than or equal to 33 IU/mL. According to the NHANES documentation (https://wwwn.cdc.gov/Nchs/Nhanes/2013-2014/SSTOXO_H.htm (accessed on 7 February 2021)) anti-*Toxoplasma gondii* concentrations were considered seronegative when less than 27 IU/mL. According to the guidelines, those values in the equivocal range defined as greater than or equal to 27 IU/mL and less than 33 IU/mL were coded as negative. In addition to the dichotomous coding of *Toxoplasma gondii* seropositivity, we also included the natural log of the IgG antibody value as a continuous measure of *Toxoplasma gondii* serointensity for analyses using the NHANES data. Before taking the natural log, we added 1 to the antibody values because the minimum value of its distribution was 0, which has an undefined natural log. The minimum antibody value of p22 and sag1 in the UK Biobank data was 1 and did not require this adjustment.

### 4.4. Covariates

We preselected covariates to include in the statistical models to adjust for possible confounding. For example, prior studies have shown associations between sociodemographic variables including age, sex, education, and income with depression [45]. Furthermore, previous studies evaluating infectious disease and depression as well as other studies in healthy adults have utilized similar covariates and have included additional variables such as smoking status, alcohol use, ethnicity, and health [33,40,46]. As such, we included covariates that possibly could affect the association between *Toxoplasma gondii* and depression. The included covariates from both the UK Biobank and NHANES are age, sex, race/ethnicity, educational attainment, income, self-rated health, body-mass index (weight in kilograms/height in meters2), smoking, and alcohol use. Some of the ways of assessing and recording the covariates differed between the UK Biobank and the NHANES: income was in pounds in the UK Biobank and in US dollars in the NHANES, the two datasets used different inventories for self-rated health, the UK Biobank used smoking status as opposed to the use of smoking frequency in the NHANES, and the UK Biobank used alcohol frequency whereas the NHANES used the number of alcoholic drinks per week. 

### 4.5. Statistical Analysis

Prior to performing the analyses, we treated missing data on the covariates using multiple imputation with chained equations. Multiple imputation uses regression-based procedures to generate multiple copies of the data with different plausible values of the missing data. The chained-equations approach allows for flexibility in modeling the distribution of the variable with missing data. In particular, nominal variables, like race-ethnicity in the NHANES dataset, can be modeled with multinomial logistic regression, while the white versus non-white variable in the UK Biobank can be modeled using logistic regression, and continuous variables like income can be modeled using linear regression. We used Stata’s mi impute command to generate 30 imputed datasets each for the UK Biobank and NHANES. Imputed datasets were separated by 200 iterations because graphical diagnostics indicated that the imputation model had converged by then [47]. All variables included in the analytic models were used in the imputation model. After the imputation process, analyses were completed using Stata’s mi estimate command, which estimates the analytic model on each of the imputed datasets separately, and then combines them into a single set of results using Rubin’s rules. 

We did parallel statistical analyses in both the UK Biobank and NHANES datasets. Because depression, the outcome variable, was binary, we used logistic regression to model the associations between *Toxoplasma gondii* seropositivity and serointensity while adjusting for the preidentified covariates. We estimated separate models for seropositivity and each measure of serointensity. We also modeled whether different levels of age, sex, educational attainment, and income affected vulnerability to depression from *Toxoplasma gondii* by interacting *Toxoplasma gondii* seropositivity and serointensity with age, sex, educational attainment, and income while adjusting for the preselected covariates. For all statistical calculations, we used Stata 17.0 (StataCorp, Stata Statistical Software, Release 17, College Station, TX, USA).

## Figures and Tables

**Table 1:** Means (proportions), Standard Deviations, Minima, and Maxima of Study Variables.

	UK Biobank		NHANES			
	Mean	SD	Mean	SD	Min	Max
Depression	0.27		0.06		0	1.00
*T. gondii*						
Seropositive	0.29		0.17		0	1.00
ln(p22)	3.42	1.37			0	8.67
ln(sag1)	4.46	0.99			0	7.41
Mean of ln(p22) and ln(sag1)	0	0.85			−0.50	2.04
IgG			0.86	1.71	0	5.32
Age (in years)	57.06	8.10	54.49	8.86	40.00	70.00
Female	0.55		0.53		0	1.00
Race-ethnicity						
Non-Hispanic white	0.93		0.42		0	1.00
Non-Hispanic black			0.19		0	1.00
Mexican American			0.15		0	1.00
Other			0.24		0	1.00
College graduate	0.39		0.55		0	1.00
Income (in 10,000 £)	4.12	3.08			0.90	12.50
Income (in $10,000)			5.85	4.11	0.25	12.50
Self-rated health	2.88	0.73			1.00	4.00
Self-rated health			3.07	0.98	1.00	5.00
Body-mass index	27.19	4.68	29.05	6.52	0	1.00
Smoking status						
Never	0.55				0	1.00
Previous	0.35				0	1.00
Current	0.10				0	1.00
Smoking frequency						
Non-smoker			0.78		0	1.00
Smokes some days			0.03		0	1.00
Smokes every day			0.18		0	1.00
Alcohol frequency						
Never	0.08				0	1.00
Daily or almost daily	0.20				0	1.00
3 or 4 times/week	0.23				0	1.00
Once or twice a week	0.26				0	1.00
1 to 3 times/month	0.11				0	1.00
Special occasions	0.12				0	1.00
Alcoholic drinks/week			2.98	7.69	0	112.00

Note: N UK Biobank = 2271. N NHANES = 2484. Results based on 30 imputed datasets. Abbreviations: NHANES = National Health and Nutrition Examination Study, SD = Standard deviation, Min = Minimum, Max = Maximum, and ln = natural log.

**Table 2:** Relationship between *Toxoplasma gondii* and Depression: Odds Ratios from Logistic Regression.

	UK Biobank				NHANES	
	Seropositive	ln(p22)	ln(sag1)	Mean of ln(p22) & ln(sag1)	Seropositive	ln(IgG)
*T. gondii*						
Seropositive	1.10				0.73	
ln(p22)		1.01				
ln(sag1)			1.04			
Mean of ln(p22) & ln(sag1)				1.05		
ln(IgG)						0.93
Age (in years)	0.96 ***	0.96 ***	0.96 ***	0.96 ***	1.01	1.01
Female	1.85 ***	1.85 ***	1.85 ***	1.85 ***	1.85 **	1.85 **
Race-ethnicity						
Non-Hispanic white	2.23 ***	2.22 ***	2.25 ***	2.24 ***	1.00	1.00
Non-Hispanic black					0.44 **	0.43 **
Mexican American					0.61	0.60
Other					0.76	0.76
College graduate	1.00	1.00	1.00	1.00	1.19	1.18
Income ^a^	0.93 ***	0.93 ***	0.93 ***	0.93 ***	0.88 ***	0.88 ***
Self-rated health	0.72 ***	0.72 ***	0.72 ***	0.72 ***	0.38 ***	0.38 ***
Body-mass index	1.02	1.02	1.02	1.02	1.01	1.01
Smoking status						
Never	1.00	1.00	1.00	1.00		
Previous	1.45 ***	1.45 ***	1.45 ***	1.45 ***		
Current	1.68 **	1.69 **	1.68 **	1.68 **		
Smoking frequency						
Non-smoker					1.00	1.00
Smokes some days					1.38	1.38
Smokes every day					1.36	1.36
Alcohol frequency						
Never	1.00	1.00	1.00	1.00		
Daily or almost daily	1.14	1.13	1.13	1.14		
3 or 4 times/week	0.88	0.87	0.87	0.88		
Once or twice a week	0.98	0.98	0.98	0.98		
1 to 3 times/month	1.24	1.24	1.24	1.25		
Special occasions	0.95	0.95	0.95	0.95		
Alcoholic drinks/week					1.01	1.01

Note: ^a^ UK income in 10,000 £; US income in $10,000. UK Biobank N = 2271. NHANES N = 2484. Results based on 30 imputed datasets. ** *p* < 0.01, *** *p* <0.001. Abbreviations: NHANES = National Health and Nutrition Examination Study, ln = natural log.

**Table 3:** Adjusted models of depression on the interaction of *T. gondii* with age, sex, education, and income: Odds ratios from regression.

	UK Biobank				NHANES	
	Seropositive	ln(p22)	ln(sag1)	Mean of ln(p22) & ln(sag1)	Seropositive	ln(IgG)
Age interaction						
*T. gondii*	0.54	1.19	1.06	0.83	5.53	1.31
Age (in years)	0.96 ***	0.97	0.96	0.96 ***	1.01	1.01
*T. gondii* x Age	1.01	1.00	1.00	1.00	0.96	0.99
Sex interaction						
*T. gondii*	1.03	0.96	0.98	1.03	0.72	0.94
Female	1.80 ***	1.42	1.24	1.85 ***	1.84 **	1.89 **
*T. gondii* x Female	1.10	1.08	1.09	1.03	1.03	0.98
Education interaction						
*T. gondii*	1.21	1.03	1.08	1.14	0.54	0.88
College degree	1.08	1.27	1.68	1.00	1.07	1.07
*T. gondii* x College degree	0.77	0.93	0.89	0.82	2.17	1.15
Income interaction						
*T. gondii*	1.07	1.01	1.07	1.02	0.96	0.99
Income ^a^	0.93 ***	0.93	0.96	0.93 ***	0.89 ***	0.89**
*T. gondii* x Income	1.01	1.00	0.99	1.01	0.92	0.98

Note: All models adjust for age, sex, race, education, income, self-rated health, body-mass index, smoking, and alcohol consumption. ^a^ UK income in 10,000 £, US income in $10,000. UK Biobank N = 2271. NHANES N = 2484. Results based on 30 imputed datasets. ** *p* < 0.01, *** *p* < 0.001. Abbreviations: NHANES = National Health and Nutrition Examination Study, ln = natural log.

## Data Availability

Data from the UK Biobank is available through application (http://www.ukbiobank.ac.uk (accessed on 7 February 2021)). Data from NHANES is available online without application and without cost (https://wwwn.cdc.gov/nchs/nhanes/continuousnhanes/default.aspx?BeginYear=2013 (accessed on 7 February 2021)).

## Supplementary Materials

The following are available online at https://www.mdpi.com/article/10.3390/pathogens10091101/s1, Table S1: NHANES full-sample relationship between *T. gondii* and Depression: Odds Ratios from Logistic Regression, Table S2: NHANES full-sample adjusted models of depression on the interaction of *T. gondii* with age, sex, education, and income: Odds ratios from logistic regression. Figure S1: Relationship between T. Gondii and Depression: Predicted Probabilities from Logistic Regression with 95% Confidence Intervals.

## References

[1] Montoya J.G., Liesenfeld O. (2004). Toxoplasmosis. Lancet.

[2] Elmore S.A., Jones J.L., Conrad P.A., Patton S., Lindsay D.S., Dubey J.P. (2010). *Toxoplasma gondii*: Epidemiology, feline clinical aspects, and prevention. Trends Parasitol..

[3] Severance E.G., Xiao J., Jones-Brando L., Sabunciyan S., Li Y., Pletnikov M., Prandovszky E., Yolken R. (2016). *Toxoplasma gondii*—A Gastrointestinal Pathogen Associated with Human Brain Diseases. Int. Rev. Neurobiol..

[4] Dalimi A., Abdoli A. (2012). Latent toxoplasmosis and human. Iran. J. Parasitol..

[5] Fallahi S., Rostami A., Nourollahpour Shiadeh M., Behniafar H., Paktinat S. (2018). An updated literature review on maternal-fetal and reproductive disorders of *Toxoplasma gondii* infection. J. Gynecol. Obstet. Hum. Reprod..

[6] Erickson L.D., Brown B.L., Gale S.D., Hedges D.W. (2021). Association between *Toxoplasma gondii* seropositivity and serointensity and brain volume in adults: A cross-sectional study. PLoS ONE.

[7] Xiao J., Yolken R.H. (2015). Strain hypothesis of *Toxoplasma gondii* infection on the outcome of human diseases. Acta Physiol..

[8] Xiao J., Kannan G., Jones-Brando L., Brannock C., Krasnova I.N., Cadet J.L., Pletnikov M., Yolken R.H. (2012). Sex-specific changes in gene expression and behavior induced by chronic Toxoplasma infection in mice. Neuroscience.

[9] Beste C., Getzmann S., Gajewski P.D., Golka K., Falkenstein M. (2014). Latent *Toxoplasma gondii* infection leads to deficits in goal-directed behavior in healthy elderly. Neurobiol. Aging.

[10] Gajewski P.D., Falkenstein M., Hengstler J.G., Golka K. (2014). *Toxoplasma gondii* impairs memory in infected seniors. Brain Behav. Immun..

[11] Gale S.D., Brown B.L., Erickson L.D., Berrett A., Hedges D.W. (2015). Association between latent toxoplasmosis and cognition in adults: A cross-sectional study. Parasitology.

[12] Gale S.D., Erickson L.D., Thacker E.L., Mitchell E.L., Brown B.L., Hedges D.W. (2020). *Toxoplasma gondii* seropositivity and serointensity and cognitive function in adults. PLoS Negl. Trop. Dis..

[13] Mendy A., Vieira E.R., Albatineh A.N., Gasana J. (2015). *Toxoplasma gondii* seropositivity and cognitive functions in school-aged children. Parasitology.

[14] Guenter W., Bielinski M., Deptula A., Zalas-Wiecek P., Piskunowicz M., Szwed K., Bucinski A., Gospodarek E., Borkowska A. (2012). Does *Toxoplasma gondii* infection affect cognitive function? A case control study. Folia Parasitol..

[15] Sugden K., Moffitt T.E., Pinto L., Poulton R., Williams B.S., Caspi A. (2016). Is *Toxoplasma gondii* Infection Related to Brain and Behavior Impairments in Humans? Evidence from a Population-Representative Birth Cohort. PLoS ONE.

[16] Torniainen-Holm M., Suvisaari J., Lindgren M., Harkanen T., Dickerson F., Yolken R.H. (2019). The lack of association between herpes simplex virus 1 or *Toxoplasma gondii* infection and cognitive decline in the general population: An 11-year follow-up study. Brain Behav. Immun..

[17] Bayani M., Riahi S.M., Bazrafshan N., Ray Gamble H., Rostami A. (2019). *Toxoplasma gondii* infection and risk of Parkinson and Alzheimer diseases: A systematic review and meta-analysis on observational studies. Acta Trop..

[18] Nayeri T., Sarvi S., Sharif M., Daryani A. (2021). *Toxoplasma gondii*: A possible etiologic agent for Alzheimer’s disease. Heliyon.

[19] Ngoungou E.B., Bhalla D., Nzoghe A., Darde M.L., Preux P.M. (2015). Toxoplasmosis and epilepsy--systematic review and meta analysis. PLoS Negl. Trop. Dis..

[20] Frye M.A., Coombes B.J., McElroy S.L., Jones-Brando L., Bond D.J., Veldic M., Romo-Nava F., Bobo W.V., Singh B., Colby C. (2019). Association of Cytomegalovirus and *Toxoplasma gondii* Antibody Titers With Bipolar Disorder. JAMA Psychiatry.

[21] Torrey E.F., Bartko J.J., Lun Z.R., Yolken R.H. (2007). Antibodies to *Toxoplasma gondii* in patients with schizophrenia: A meta-analysis. Schizophr. Bull..

[22] Sutterland A.L., Fond G., Kuin A., Koeter M.W., Lutter R., van Gool T., Yolken R., Szoke A., Leboyer M., de Haan L. (2015). Beyond the association. *Toxoplasma gondii* in schizophrenia, bipolar disorder, and addiction: Systematic review and meta-analysis. Acta Psychiatr. Scand..

[23] Al-Hussainy N.H., Al-Saedi A.M., Al-Lehaibi J.H., Al-Lehaibi Y.A., Al-Sehli Y.M., Afifi M.A. (2015). Serological evidences link toxoplasmosis with schizophrenia and major depression disorder. J. Microsc. Ultrastruct..

[24] Duffy A.R., Beckie T.M., Brenner L.A., Beckstead J.W., Seyfang A., Postolache T.T., Groer M.W. (2015). Relationship Between *Toxoplasma gondii* and Mood Disturbance in Women Veterans. Mil. Med..

[25] Kamal A.M., Kamal A.M., Abd El-Fatah A.S., Rizk M.M., Hassan E.E. (2020). Latent Toxoplasmosis is Associated with Depression and Suicidal Behavior. Arch. Suicide Res..

[26] Nasirpour S., Kheirandish F., Fallahi S. (2020). Depression and *Toxoplasma gondii* infection: Assess the possible relationship through a seromolecular case-control study. Arch. Microbiol..

[27] Nourollahpour Shiadeh M., Rostami A., Pearce B.D., Gholipourmalekabadi M., Newport D.J., Danesh M., Mehravar S., Seyyedtabaei S.J. (2016). The correlation between *Toxoplasma gondii* infection and prenatal depression in pregnant women. Eur. J. Clin. Microbiol. Infect. Dis..

[28] Yalin Sapmaz S., Sen S., Ozkan Y., Kandemir H. (2019). Relationship between *Toxoplasma gondii* seropositivity and depression in children and adolescents. Psychiatry Res..

[29] Alvarado-Esquivel C., Martinez-Martinez A.L., Sanchez-Anguiano L.F., Hernandez-Tinoco J., Castillo-Orona J.M., Salas-Martinez C., Sifuentes-Alvarez A., Sandoval-Carrillo A.A., Salas-Pacheco J.M., Liesenfeld O. (2017). Lack of association between *Toxoplasma gondii* exposure and depression in pregnant women: A case-control study. BMC Infect. Dis..

[30] Chen X., Chen B., Hou X., Zheng C., Yang X., Ke J., Hu X., Tan F. (2019). Association between *Toxoplasma gondii* infection and psychiatric disorders in Zhejiang, Southeastern China. Acta Trop..

[31] Flegr J., Hodny Z. (2016). Cat scratches, not bites, are associated with unipolar depression--cross-sectional study. Parasites Vectors.

[32] Gale S.D., Brown B.L., Berrett A., Erickson L.D., Hedges D.W. (2014). Association between latent toxoplasmosis and major depression, generalised anxiety disorder and panic disorder in human adults. Folia Parasitol..

[33] Pearce B.D., Kruszon-Moran D., Jones J.L. (2012). The relationship between *Toxoplasma gondii* infection and mood disorders in the third National Health and Nutrition Survey. Biol. Psychiatry.

[34] Holuka C., Merz M.P., Fernandes S.B., Charalambous E.G., Seal S.V., Grova N., Turner J.D. (2020). The COVID-19 Pandemic: Does Our Early Life Environment, Life Trajectory and Socioeconomic Status Determine Disease Susceptibility and Severity?. Int. J. Mol. Sci..

[35] Alvarado-Esquivel C., Sanchez-Anguiano L.F., Hernandez-Tinoco J., Berumen-Segovia L.O., Torres-Prieto Y.E., Estrada-Martinez S., Perez-Alamos A.R., Ortiz-Jurado M.N., Molotla-de-Leon G., Beristain Garcia I. (2016). *Toxoplasma gondii* Infection and Mixed Anxiety and Depressive Disorder: A Case-Control Seroprevalence Study in Durango, Mexico. J. Clin. Med. Res..

[36] Lehmann T., Marcet P.L., Graham D.H., Dahl E.R., Dubey J.P. (2006). Globalization and the population structure of *Toxoplasma gondii*. Proc. Natl. Acad. Sci. USA.

[37] Xiao J., Buka S.L., Cannon T.D., Suzuki Y., Viscidi R.P., Torrey E.F., Yolken R.H. (2009). Serological pattern consistent with infection with type I *Toxoplasma gondii* in mothers and risk of psychosis among adult offspring. Microbes Infect..

[38] Bay-Richter C., Petersen E., Liebenberg N., Elfving B., Wegener G. (2019). Latent toxoplasmosis aggravates anxiety- and depressive-like behaviour and suggest a role of gene-environment interactions in the behavioural response to the parasite. Behav. Brain Res..

[39] Sudlow C., Gallacher J., Allen N., Beral V., Burton P., Danesh J., Downey P., Elliott P., Green J., Landray M. (2015). UK biobank: An open access resource for identifying the causes of a wide range of complex diseases of middle and old age. PLoS Med..

[40] Smith D.J., Nicholl B.I., Cullen B., Martin D., Ul-Haq Z., Evans J., Gill J.M., Roberts B., Gallacher J., Mackay D. (2013). Prevalence and characteristics of probable major depression and bipolar disorder within UK biobank: Cross-sectional study of 172,751 participants. PLoS ONE.

[41] Spitzer R.L., Kroenke K., Williams J.B. (1999). Validation and utility of a self-report version of PRIME-MD: The PHQ primary care study. Primary Care Evaluation of Mental Disorders. Patient Health Questionnaire. JAMA.

[42] Kroenke K., Spitzer R.L., Williams J.B.W. (2001). The PHQ-9: Validity of a brief depression severity measure. J. Gen. Intern. Med..

[43] Kroenke K., Spitzer R.L. (2002). The PHQ-9: A new depression diagnostic and severity measure. Psychiatr. Ann..

[44] Brenner N., Mentzer A.J., Butt J., Braband K.L., Michel A., Jeffery K., Klenerman P., Gartner B., Schnitzler P., Hill A. (2019). Validation of Multiplex Serology for human hepatitis viruses B and C, human T-lymphotropic virus 1 and *Toxoplasma gondii*. PLoS ONE.

[45] Akhtar-Danesh N., Landeen J. (2007). Relation between depression and sociodemographic factors. Int. J. Ment. Health Syst..

[46] Gale S.D., Berrett A.N., Erickson L.D., Brown B.L., Hedges D.W. (2018). Association between virus exposure and depression in US adults. Psychiatry Res..

[47] Enders C.K. (2010). Applied Missing Data Analysis.

