# Supplementary material for: Examining the Relationship between Toxoplasma gondii and Seropositivity and Serointensity and Depression in Adults from the United Kingdom and the United States: A Cross-Sectional Study
Source: Pathogens. 2021 Aug 29;10(9):1101. doi: 10.3390/pathogens10091101 (PMC8470860; doi:10.3390/pathogens10091101)
Supplement: Supplementary file 1 [file pathogens-10-01101-s001.zip › pathogens-1305378-supplementary.pdf]

Table S1. NHANES full-sample relationship between *T. gondii* and Depression:.Odds Ratios from Logistic Regression.

	Seropositive	ln(IgG)
T. gondii		
Seropositive	0.72	
ln(IgG)		0.93
Age (in years)	1.00	1.00
Female	1.58**	1.58**
Race-ethnicity		
Non-Hispanic white	1.00	1.00
Non-Hispanic black	0.54**	0.54**
Mexican American	0.68	0.68
Other	0.89	0.90
College graduate	1.04	1.04
Income (in $10,000)	0.91***	0.91***
Self-rated health	0.40***	0.40***
Body-mass index	1.02*	1.02*
Smoking frequency		
Non-smoker	1.00	1.00
Smokes some days	1.37	1.37
Smokes every day	1.73***	1.73**
Alcoholic drinks/week	1.01	1.01

Note: N = 5,028. Results based on 30 imputed datasets. * $p < 0.05$, ** $p < 0.01$, *** $p < 0.001$. Abbreviations: NHANES = National Health and Nutrition Examination Study, ln = natural log.

Table S2. NHANES full-sample adjusted models of depression on the interaction of *T. gondii* with age, sex, education, and income: Odds ratios from logistic regression.

	Seropositive	ln(IgG)
Age Interaction		
T. gondii	0.55	0.82
Age (in years)	1.00	1.00
T. gondii x Age	1.00	1.00
Sex interaction		
T. gondii	0.70	0.94
Female	1.58**	1.60**
T. gondii x Female	1.03	0.99
Education interaction		
T. gondii	0.53*	0.88*
College degree	0.94	0.94
T. gondii x College degree	2.17	1.15
Income interaction		
T. gondii	0.98	0.99
Income (in $10,000)	0.92***	0.92**
T. gondii x Income	0.90	0.98

Note: N = 5,028. All models adjust for age, gender, race, education, income, self-rated health, body-mass index, smoking, and alcohol consumption. Results based on 30 imputed datasets. * $p < 0.05$, ** $p < 0.01$, *** $p < .001$. Abbreviations: NHANES = National Health and Nutrition Examination Study, ln = natural log.

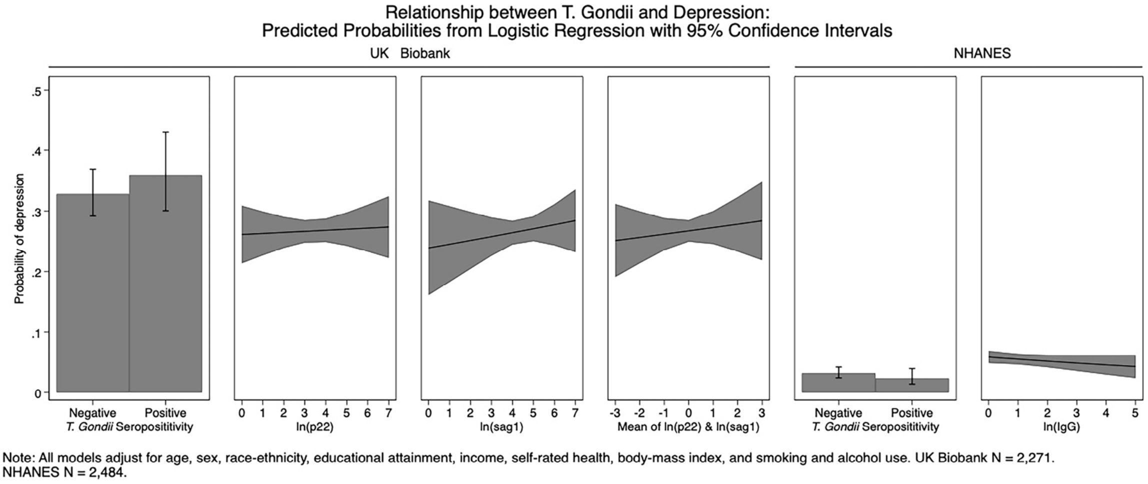

Figure S1. .Relationship between T. Gondii and Depression: Predicted Probabilities from Logistic Regression with 95% Confidence Intervals

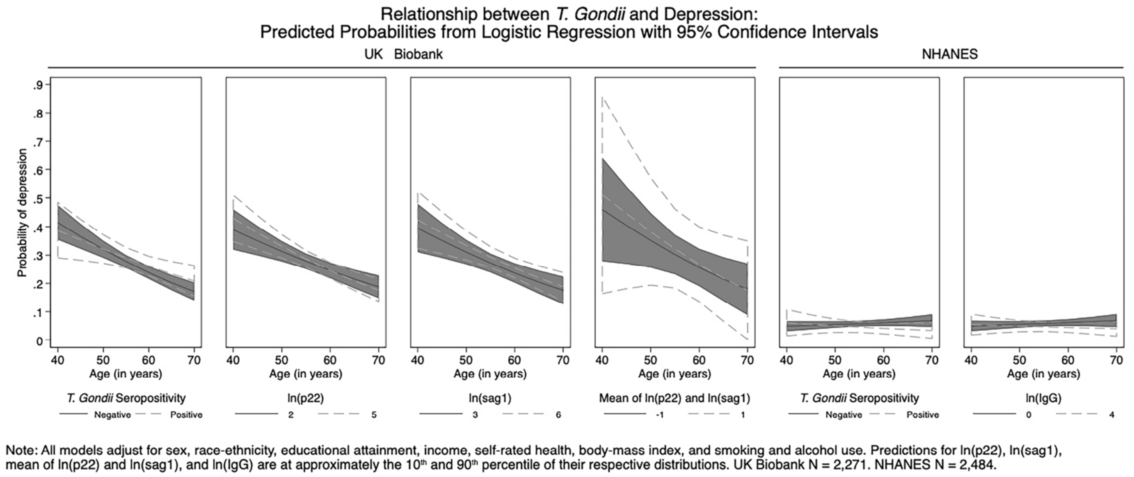

(**a**)

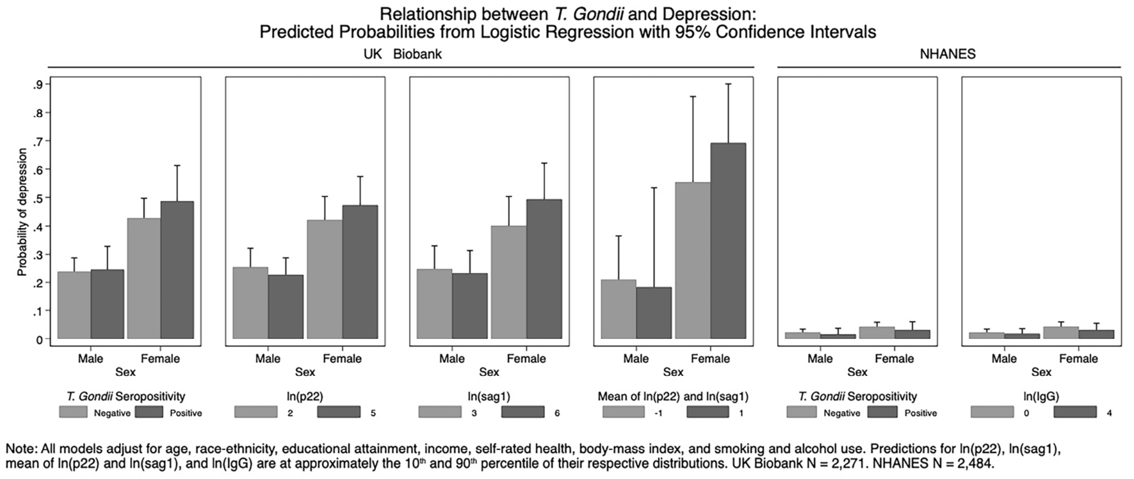

(**b**)

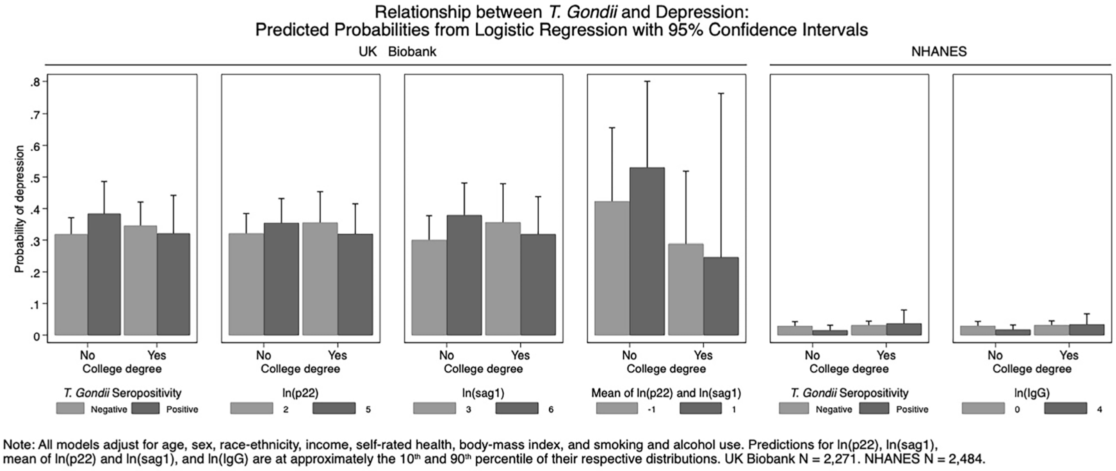
Relationship between T. Gondii and Depression:
Predicted Probabilities from Logistic Regression with 95% Confidence Intervals
UK Biobank
NHANES
Probability of depression
0
.1
.2
.3
.4
.5
.6
.7
.8
No
Yes
College degree
T. Gondii Seropositivity
Negative
Positive
ln(p22)
2
5
ln(sag1)
3
6
Mean of ln(p22) and ln(sag1)
-1
1
ln(IgG)
0
4
Note: All models adjust for age, sex, race-ethnicity, income, self-rated health, body-mass index, and smoking and alcohol use. Predictions for ln(p22), ln(sag1),
mean of ln(p22) and ln(sag1), and ln(IgG) are at approximately the 10th and 90th percentile of their respective distributions. UK Biobank N = 2,271. NHANES N = 2,484.

(**c**)

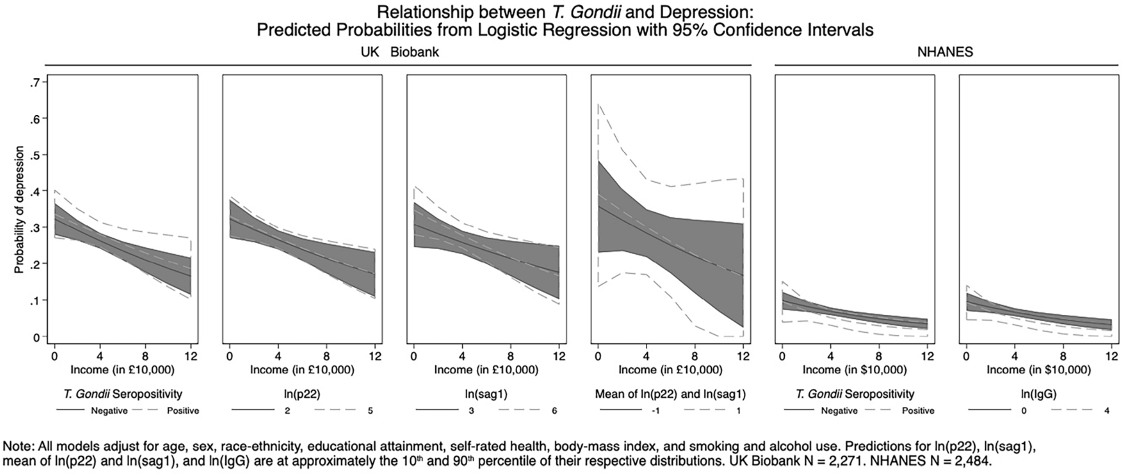
Relationship between T. Gondii and Depression:
Predicted Probabilities from Logistic Regression with 95% Confidence Intervals
UK Biobank
NHANES
Probability of depression
0
.1
.2
.3
.4
.5
.6
.7
0
4
8
12
Income (in £10,000)
Income (in $10,000)
T. Gondii Seropositivity
Negative
Positive
ln(p22)
2
5
ln(sag1)
3
6
Mean of ln(p22) and ln(sag1)
-1
1
ln(IgG)
0
4
Note: All models adjust for age, sex, race-ethnicity, educational attainment, self-rated health, body-mass index, and smoking and alcohol use. Predictions for ln(p22), ln(sag1),
mean of ln(p22) and ln(sag1), and ln(IgG) are at approximately the 10th and 90th percentile of their respective distributions. UK Biobank N = 2,271. NHANES N = 2,484.

(**d**)

Figure S2. .
